# Predictors for Mild and Severe Hypoglycemia in Insulin-Treated Japanese Diabetic Patients

**DOI:** 10.1371/journal.pone.0130584

**Published:** 2015-06-23

**Authors:** Nao Sonoda, Akiko Morimoto, Satoshi Ugi, Katsutaro Morino, Osamu Sekine, Ken-ichi Nemoto, Kayo Godai, Hiroshi Maegawa, Naomi Miyamatsu

**Affiliations:** 1 Department of Clinical Nursing, Shiga University of Medical Science, Otsu, Shiga, Japan; 2 Department of Medicine, Shiga University of Medical Science, Otsu, Shiga, Japan; Baylor College of Medicine, UNITED STATES

## Abstract

The objective of this study was to explore predictors, including social factors, lifestyle factors, and factors relevant to glycemic control and treatment, for mild and severe hypoglycemia in insulin-treated Japanese diabetic patients. This study included 123 insulin-treated diabetic patients who were referred to the diabetes clinic between January and July 2013 at Shiga University of Medical Science Hospital. After a survey examining the various factors, patients were followed for 6 months. During the follow-up period, blood glucose was self-monitored. Mild hypoglycemia was defined as blood glucose level 50–69 mg/dl, and severe hypoglycemia was defined as blood glucose level ≤49 mg/dl. Multinomial logistic regression was used to estimate the adjusted odds ratio (OR) and 95% confidence interval (CI) of each factor for mild and severe hypoglycemia. During the 6-month follow-up period, 41 (33.3%) patients experienced mild hypoglycemia, and 20 (16.3%) experienced severe hypoglycemia. In multivariable-adjusted analyses, assistance from family members at the time of the insulin injection [presence/absence, OR (95% CI): 0.39 (0.16–0.97)] and drinking [current drinker/non- and ex-drinker, OR (95% CI): 4.89 (1.68–14.25)] affected mild hypoglycemia. Assistance from family members at the time of insulin injection [presence/absence, OR (95% CI): 0.19 (0.05–0.75)] and intensive insulin therapy [yes/no, OR (95% CI): 3.61 (1.06–12.26)] affected severe hypoglycemia. In conclusion, our findings suggest that not only a factor relevant to glycemic control and treatment (intensive insulin therapy) but also a social factor (assistance from family members) and a lifestyle factor (current drinking) were predictors for mild or severe hypoglycemia in Japanese insulin-treated diabetic patients.

## Introduction

Hypoglycemia is one of the most undesirable and unpredictable side-effects in insulin-treated diabetic patients. In recent years, it has been reported that severe hypoglycemia is associated with a higher risk of cardiovascular disease and dementia [1,2]. Additionally, even mild hypoglycemia is associated with reduced quality of life [3]. Repetitive mild hypoglycemia induces a state of hypoglycemic tolerance, where symptomatic and counter-regulatory responses are elicited at low blood glucose levels [4,5]. Therefore, it is important for patients with insulin-treated diabetes to prevent mild as well as severe hypoglycemia, and to identify predictors for mild and severe hypoglycemia.

Many previous studies have reported episodes of hypoglycemia in hospital emergency departments [6–8]. However, most episodes of hypoglycemia are treated effectively at home or at work by relatives, friends, or colleagues and do not require the assistance of emergency medical services [9]. Cases treated in the emergency department are recognized to represent the tip of the iceberg. Additionally, hypoglycemia is often evaluated by self-reported symptoms without confirmation by blood glucose measurement [10–12]. However, hypoglycemia is difficult to assess with accuracy, unless measured, because self-reported symptoms of hypoglycemia may be symptoms of other diseases such as hypotension, anemia, and menopausal disorders. Moreover, the retrospective recall of symptomatic hypoglycemia has been shown to be inaccurate beyond an interval of 1 week [13]. Therefore, to identify predictors for mild and severe hypoglycemia, it is necessary to determine hypoglycemic episodes using blood glucose measurements in hospital patient departments.

Regarding predictors for hypoglycemia, insulin use and intensive therapy are most consistently and strongly associated with risk for severe hypoglycemia in patients with diabetes [14–16]. Additionally, it has been reported that use of sulfonylureas and impaired renal function are associated with hypoglycemia [17,18]. However, it may not be possible to easily modify these factors relevant to glycemic control and treatment. Therefore, it is important to identify modifiable factors for mild and severe hypoglycemia, but little attention has been given to social and lifestyle factors [19,20]. Because of the importance of investigating these various factors, we explore predictors, including social factors, lifestyle factors, and factors relevant to glycemic control and treatment, for mild and severe hypoglycemia among insulin-treated Japanese diabetic patients, with hypoglycemia determined using blood glucose measurements.

## Materials and Methods

### Study participants

This study included 195 insulin-treated diabetic patients who were referred to a diabetes clinic between January 2013 and July 2013 at Shiga University of Medical Science Hospital (Otsu, Japan). The exclusion criteria were patients less than 20 years of age, those with dementia, or those with gestational diabetes. Of 195 insulin-treated diabetic patients, 185 (94.9%) patients agreed to participate in the survey. Of these 185 patients, we excluded 21 with missing data. After a survey examining the various factors, 123 patients completed a 6-month follow-up and were included in the analysis.

### Ethics Statement

The approval for this study was obtained from the Institutional Review Board of Shiga University of Medical Science (No. 24-141-2, 2012), and the participants gave their written informed consent.

### Procedures

A survey examining the various factors was conducted between January 2013 and July 2013. Demographic characteristics, social factors, and lifestyle factors were obtained using a self-administered questionnaire that was partially supported by a personal interview with nurses. The social factors measured included: education, occupation, living arrangement, and assistance from family members for insulin injections. Education was categorized as ≤12 years or >12 years. Living arrangement was categorized as living together or living alone. Both occupation and assistance from family members at the insulin injection were categorized as presence or absence. The lifestyle factors measured included: smoking status, drinking status, meal regularity, and exercise habits. Smoking status was categorized as current, non-, or ex-smoker, and drinking status was categorized as current, non-, or ex-drinker. Meal regularity was categorized as regular (fixed meal times) or irregular (irregular meal times or skipped breakfast, lunch or dinner). Exercise habit was categorized as yes or no.

Factors relevant to glycemic control and treatment, including daily dosage and types of insulin, use of sulfonylureas and glinides, were collected by a review of the patients’ medical records. Intensive insulin therapy was defined as use of a combination of rapid/short-acting insulin and long/intermediate-acting insulin. Hemoglobin A1c (HbA1c) levels and estimated glomerular filtration rate levels were collected by a review of the patients’ most recent medical records. HbA1c (%) was estimated as a National Glycohemoglobin Standardization Program equivalent value (%) and calculated using the formula HbA1c (%) = 1.02 × HbA1c (Japan Diabetes Society, %) + 0.25% [21].

The patients were weighed while wearing light clothing, and height was measured without shoes. Blood pressure was measured by trained nurses using an automatic sphygmomanometer, with the patients in the sitting position after resting for at least 5 min.

### Definition of hypoglycemia

After the survey examining the various factors, patients were followed-up for 6 months. During the follow-up period, patients performed self-monitoring of blood glucose. They measured blood glucose regularly (approximately 2–4 times per day) and at times when they felt hypoglycemic. They were not required to deviate from their normal routine of blood glucose monitoring. In this study, hypoglycemia was defined as a blood glucose level of <70 mg/dl [22]. It has been reported that the glycemic threshold for neurogenic and neuroglycopenic symptoms and cognitive impairments is ~50–55 mg/dl [23]. In this study, mild hypoglycemia was defined as a blood glucose level of 50–69 mg/dl, and severe hypoglycemia was defined as a blood glucose level of ≤49 mg/dl.

### Statistical analysis

The proportion of patients who experienced mild and severe hypoglycemia according to each factor was compared using the χ^2^ test or Fisher’s exact test. Additionally, in order to confirm the predictors for mild and severe hypoglycemia, adjusted odds ratios (ORs) and 95% confidence intervals (CIs) for mild and severe hypoglycemia according to each factor were calculated using multinomial logistic regression analysis (response variable: 1 = patients who did not experience hypoglycemia, 2 = patients who experienced only mild hypoglycemia, and 3 = patients who experienced severe hypoglycemia). All data were analyzed using SPSS statistical software (version 21.0J; IBM SPSS Japan, Tokyo, Japan). All reported *p* values are two-tailed; values <0.05 were considered statistically significant.

## Results

Characteristics of the participants are shown in Table 1. Their mean age was 65.9 years and their mean HbA1c level was 7.8%.

**Table 1:** Characteristics of 123 Japanese patients with insulin-treated diabetes.

Variable	Number (%) / Mean ± standard deviation
Age (years)	65.9 ± 12.5
Men, n (%)	70 (56.9)
Diabetes duration (years)	20.4 ± 10.3
Type 1 diabetes, n (%)	25 (20.3)
Body mass index (kg/m^2^)	25.0 ± 4.0
Systolic blood pressure (mmHg)	136.3 ± 18.5
Diastolic blood pressure (mmHg)	72.4 ± 10.7
Education >12 years, n (%)	55 (44.7)
Having an occupation, n (%)	69 (56.1)
Living together, n (%)	115 (93.5)
Presence of assistance from family members at the insulin injection, n (%)	46 (37.4)
Current smoker, n (%)	19 (15.4)
Current drinker, n (%)	39 (31.7)
Regular meal, n (%)	96 (78.0)
Exercise habit, n (%)	59 (48.0)
HbA1c (%)	7.8 ± 1.0
eGFR (ml/min/1.73 m^2^)	69.2 ± 24.0
Total daily insulin (U/kg)	0.5 ± 0.3
Insulin type, n (%)	
Rapid-acting + long-acting	63 (51.2)
Rapid-acting + intermediate-acting	1 (0.8)
Short-acting + long-acting	1 (0.8)
Rapid-acting + pre-mixed	4 (3.3)
Pre-mixed + pre-mixed	1 (0.8)
Rapid-acting	6 (4.9)
Pre-mixed	27 (22.0)
Long-acting	20 (16.3)

HbA1c, hemoglobin A1c; eGFR, estimated glomerular filtration rate.

Of the 123 patients, 61 (49.6%) patients experienced hypoglycemia (blood glucose level <70 mg/dl) during the 6-month follow-up period. Of these 61 patients, 41 (33.3%) experienced only mild hypoglycemia, and 20 (16.3%) experienced severe hypoglycemia.

Table 2 shows the proportion of patients who experienced mild and severe hypoglycemia according to each factor during the 6-month follow-up period. Current drinkers had a 1.6-fold (54.8% vs. 33.3%, *p* = 0.041) higher proportion of patients who experienced mild hypoglycemia, compared with non- and ex-drinkers. On the other hand, those who received assistance from family members with the insulin injection had a 0.5-fold (25.6% vs. 50.0%, *p* = 0.013) proportion of patients who experienced mild hypoglycemia, compared with those with no family assistance. In addition, those who received intensive insulin therapy had a 3.0-fold (36.6% vs. 12.2%, *p* = 0.010) higher proportion of patients who experienced severe hypoglycemia, compared with those who did not receive intensive insulin therapy. On the other hand, those who received assistance from family members with the insulin injection had a 0.2-fold (8.6% vs. 36.2%, *p* = 0.004) proportion of patients who experienced severe hypoglycemia, compared with those with no family assistance.

**Table 2:** Proportion of patients who experienced mild and severe hypoglycemia by each factor during the 6-month follow-up period.

	Proportion of patients who experienced mild hypoglycemia (50–69 mg/dl), % (incident case/n)	*p* value	Proportion of patients who experienced severe hypoglycemia (≤49 mg/dl), % (incident case/n)	*p* value
n	103^a^		82^b^	
**Social factors**				
Education: ≤12 years	43.1 (25/58)	0.438	25.6 (10/39)	0.802
Education: >12 years	35.6 (16/45)		23.3 (10/43)	
Occupation: presence	41.1 (23/56)	0.775	28.3 (13/46)	0.356
Occupation: absence	38.3 (18/47)		19.4 (7/36)	
Living arrangement: living together	39.6 (38/96)	1.000	24.7 (19/77)	1.000
Living arrangement: living alone	42.9 (3/7)		20.0 (1/5)	
Assistance from family members at the insulin injection: presence	25.6 (11/43)	0.013	8.6 (3/35)	0.004
Assistance from family members at the insulin injection: absence	50.0 (30/60)		36.2 (17/47)	
**Lifestyle factors**				
Smoking: current smoker	40.0 (6/15)	0.987	30.8 (4/13)	0.725
Smoking: non- and ex-smoker	39.8 (35/88)		23.2 (16/69)	
Drinking: current drinker	54.8 (17/31)	0.041	36.4 (8/22)	0.126
Drinking: non- and ex-drinker	33.3 (24/72)		20.0 (12/60)	
Meal regularity: regular	43.0 (34/79)	0.224	27.4 (17/62)	0.373
Meal regularity: irregular	29.2 (7/24)		15.0 (3/20)	
Exercise habits: yes	34.6 (18/52)	0.277	17.1 (7/41)	0.123
Exercise habits: no	45.1 (23/51)		31.7 (13/41)	
**Factors relevant to glycemic control and treatment**				
HbA1c: ≤6.9%	31.8 (7/22)	0.338	28.6 (6/21)	0.779
HbA1c: 7.0–7.9%	48.7 (19/39)		25.9 (7/27)	
HbA1c: ≥8.0%	35.7 (15/42)		20.6 (7/24)	
eGFR: ≥90 ml/min/1.73 m^2^	42.9 (9/21)	0.395	20.0 (3/15)	0.328
eGFR: 60–89 ml/min/1.73 m^2^	44.9 (22/49)		25.0 (9/36)	
eGFR: <60 ml/min/1.73 m^2^	30.3 (10/33)		25.8 (8/31)	
Total daily insulin: ≤0.29 U/kg	37.0 (10/27)	0.896	19.0 (4/21)	0.582
Total daily insulin: 0.30–0.49 U/kg	38.9 (14/36)		21.4 (6/28)	
Total daily insulin: ≥0.50 U/kg	42.5 (17/40)		30.3 (10/33)	
Intensive insulin therapy: yes (rapid-/short-acting and long-/intermediate-acting insulin)	48.0 (24/50)	0.099	36.6 (15/41)	0.010
Intensive insulin therapy: no (other insulin type)	32.1 (17/53)		12.2 (5/41)	
Use of the sulfonylureas: yes	27.3 (3/11)	0.519	11.1 (1/9)	0.442
Use of the sulfonylureas: no	41.3 (38/92)		26.0 (19/73)	
Use of the glinide: yes	50.0 (5/10)	0.514	0.0 (0/5)	0.328
Use of the glinide: no	38.7 (36/93)		26.0 (20/77)	

Dichotomous and categorical data are analyzed by χ^2^ test and Fisher’s exact test, and are shown as % (incident case/n).

^a^Excluded 20 patients who experienced severe hypoglycemia.

^b^Excluded 41 patients who experienced only mild hypoglycemia.

HbA1c, hemoglobin A1c; eGFR, estimated glomerular filtration rate.

Table 3 shows ORs and 95% CIs for mild and severe hypoglycemia according to each factor. In multivariable-adjusted analyses, assistance from family members at the time of the insulin injection [presence/absence, OR (95% CI): 0.39 (0.16–0.97)] and drinking [current drinker/non- and ex-drinker, OR (95% CI): 4.89 (1.68–14.25)] affected mild hypoglycemia. Similarly, assistance from family members at the time of insulin injection [presence/absence, OR (95% CI): 0.19 (0.05–0.75)] and those on intensive insulin therapy [yes/no, OR (95% CI): 3.61 (1.06–12.26)] affected severe hypoglycemia. These results were the same when we adjusted for all factors using the multivariable-adjusted model plus the frequency of self-measurement of blood glucose. Although the statistical power decreased because the sample size was low, the tendency of these results did not change after stratification by sex and age (<60 years or ≥60 years).

**Table 3:** Odds ratios and 95% confidence intervals for mild and severe hypoglycemia according to each factor in 123 patients with insulin-treated diabetes during the 6-month follow-up period.

Explanatory variable	Mild hypoglycemia (50–69 mg/dl)	Severe hypoglycemia (≤49 mg/dl)
**Social factors**		
Assistance from family members at the insulin injection (presence/absence)		
Age- and sex-adjusted OR (95% CI)	0.37 (0.16–0.88)	0.18 (0.05–0.69)
Multivariable-adjusted OR (95% CI)^a^	0.39 (0.16–0.97)	0.19 (0.05–0.75)
**Lifestyle factors**		
Drinking (current drinker/non- and ex-drinker)		
Age- and sex-adjusted OR (95% CI)	5.07 (1.79–14.37)	3.08 (0.94–10.10)
Multivariable-adjusted OR (95% CI)^a^	4.89 (1.68–14.25)	2.76 (0.78–9.80)
**Factors relevant to glycemic control and treatment**		
Intensive insulin therapy (yes/no)		
Age- and sex-adjusted OR (95% CI)	1.64 (0.71–3.82)	3.72 (1.15–12.03)
Multivariable-adjusted OR (95% CI)^a^	1.63 (0.67–4.01)	3.61 (1.06–12.26)

Multinomial logistic regression was used to estimate the adjusted OR and 95% CI for mild and severe hypoglycemia.

Response variable: 1 = patients who did not experience hypoglycemia, 2 = patients who experienced only mild hypoglycemia, and 3 = patients who experienced severe hypoglycemia.

^a^Adjusted for age, sex, assistance from family members at the insulin injection (presence or absence), drinking (current drinker or non- and ex-drinker), and intensive insulin therapy (yes or no).

OR, odds ratio; CI, confidence interval.

## Discussion

This study indicates that lack of assistance from family members at the time of insulin injection and receiving intensive insulin therapy were predictors for severe hypoglycemia. Additionally, lack of assistance from family members at the time of the insulin injection and current drinking were predictors for mild hypoglycemia. In other words, our findings identified correctable factors (i.e., assistance from family members and current drinking) that exist for the prevention of hypoglycemia in insulin treated diabetic patients.

Severe hypoglycemia is a potentially life-threatening condition that can cause seizures, loss of consciousness, brain damage, dementia, and even death. Additionally, even mild hypoglycemia is an undesirable side effect. Therefore, it is important to prevent hypoglycemia. In the present study, we found that assistance from family members at the time of insulin injection was associated with a reduced risk for both mild and severe hypoglycemia, after adjustment for intensive insulin therapy. In previous studies, support from family members was associated with adherence to diabetes treatment [24] and self-care behaviors [25]. Similarly, in the present study, assistance from family members at the time of insulin injection may have led to better adherence and behavior of patients. This is an important factor to consider because the mean age of patients in this study was 66 years, and elderly patients may require more assistance to adhere to treatments. In addition, there is a possibility that mistakes in insulin injections might be prevented by confirming the dosage or the time of the insulin injection together by the patients and their family members. Notably, 38 (92.7%) of 41 patients who experienced only mild hypoglycemia and 19 (95.0%) of 20 patients who experienced severe hypoglycemia were ‘living together’, i.e., not living alone (S1 Table). However, only 11 (26.8%) of 41 patients who experienced mild hypoglycemia and only 3 (15.0%) of 20 patients who experienced severe hypoglycemia had assistance from family members at insulin injections (S1 Table). To increase the role of family members in supportive behaviors for the prevention of hypoglycemia, medical workers should consider involving family members in the management of patients with diabetes, especially for elderly patients with diabetes.

Current alcohol drinking was associated with a higher risk of mild hypoglycemia, and was the only lifestyle factor associated with hypoglycemia in this study. Alcohol has glucose-lowering effects and can mask symptoms of hypoglycemia. In the present study, 17 (41.5%) of 41 patients who experienced mild hypoglycemia and 8 (40.0%) of 20 patients who experienced severe hypoglycemia were current drinkers (S1 Table). Therefore, it is necessary for the management team to confirm the patients’ knowledge of the impact of alcohol on hypoglycemia, and to advise if appropriate on giving up drinking for prevention of hypoglycemia. In this study, current drinking was not associated with severe hypoglycemia, and some drinkers who had previously experienced severe hypoglycemia made the decision to give up drinking.

Intensive insulin therapy was associated with a higher risk of severe hypoglycemia in this study, and this finding is in agreement with previous reports [14,15]. In short, patients who receive intensive insulin therapy are a high-risk group for future hypoglycemic incidents. In the present study, only 21 (32.3%) of 65 patients who received intensive insulin therapy had assistance from family members for their insulin injection, and 20 (30.8%) of 65 patients who received intensive insulin therapy were current drinkers (data not shown). It is important that physicians not only consider the daily quantity or type of insulin, but should also intervene in the modifiable factors that affect hypoglycemia among patients who are at high risk for future hypoglycemic incidents.

There are several limitations to the present study. First, the participants were limited to the patients of one university hospital. Second, the calculated 95% CIs were wide because of the small number of incident cases in some groups. Third, we did not separately assess predictors for mild and severe hypoglycemia in types 1 and 2 diabetes, because the sample size of patients with type 1 diabetes was low. However, the type of diabetes did not affect the risk of hypoglycemia in this study (data not shown). Fourth, the definition of severe hypoglycemia of the American Diabetes Association (ADA) is “An event requiring assistance of another person to actively administer carbohydrate, glucagons, or other resuscitative actions” [26]. It is very likely that patients with severe hypoglycemia have a blood glucose level of ≤49 mg/dl, but not all patients with a blood glucose level ≤49 mg/dl may have severe hypoglycemia according to the ADA definition. Consequently, the proportion of patients experiencing severe hypoglycemia may be overestimated in this study. Unfortunately, we did not evaluate the cases of severe hypoglycemia using the ADA criteria, and therefore, we were not able to calculate the proportion of patients meeting this definition. Hence, a further investigation that evaluates the incidences of severe hypoglycemia based on the ADA criteria is necessary to confirm our results. Finally, of 185 patients who agreed to participate in the survey, 62 patients (21 patients with missing data and 41 patients who did not complete the 6-month follow-up) were excluded from our analysis. However, the characteristics such as age and HbA1c level did not differ significantly between the included and excluded patients in our analyses (S2 Table).

In conclusion, to our knowledge, this is the first report to suggest that not only a factor relevant to glycemic control and treatment (intensive insulin therapy) but also a social factor (assistance from family members at the time of the insulin injection) and a lifestyle factor (current drinking) were predictors for mild or severe hypoglycemia in Japanese insulin-treated diabetic patients. It is important that medical workers educate not only patients but also their family members; and appropriate intervention should be taken with patients who drink.

## Supporting Information

S1 Table Characteristics of patients who either did not experience hypoglycemia, who experienced only mild hypoglycemia, or who experienced severe hypoglycemia. Note: Continuous data were analyzed by analysis of covariance with adjustments for age and sex, and are shown as age- and sex-adjusted mean (95% confidence interval). Dichotomous data were analyzed by χ^2^ test and are shown as number (%). HbA1c, hemoglobin A1c; eGFR, estimated glomerular filtration rate.
(PDF)

S2 Table Comparison between the characteristics of patients who were included and excluded from our analysis among all the patients who agreed to participate in the survey. Note: Continuous data were analyzed by analysis of covariance with adjustments for age and sex, and are shown as age- and sex-adjusted mean (95% confidence interval). Dichotomous data were analyzed by χ^2^ test, and are shown as number (%). HbA1c, hemoglobin A1c; eGFR, estimated glomerular filtration rate.
(PDF)
