# Supplementary material for: Predictors for Mild and Severe Hypoglycemia in Insulin-Treated Japanese Diabetic Patients
Source: PLoS One. 2015 Jun 23;10(6):e0130584. doi: 10.1371/journal.pone.0130584 (PMC4477874; doi:10.1371/journal.pone.0130584)
Supplement: S1 Table — Note: Continuous data were analyzed by analysis of covariance with adjustments for age and sex, and are shown as age- and sex-adjusted mean (95% confidence interval). Dichotomous data were analyzed by χ2 test and are shown as number (%). HbA1c, hemoglobin A1c; eGFR, estimated glomerular filtration rate. (PDF) [file pone.0130584.s001.pdf]

S1 Table. Characteristics of patients who either did not experience hypoglycemia, who experienced only mild hypoglycemia, or who experienced severe hypoglycemia.

	Patients who did not experience hypoglycemia	Patients who experienced only mild hypoglycemia	Patients who experienced severe hypoglycemia	*p* value
n	62	41	20	
Age (years)	65.6 (62.6–68.7)	65.5 (61.8–69.2)	67.6 (61.7–73.5)	0.830
Men, n (%)	40 (64.5)	18 (43.9)	12 (60.0)	0.113
Education >12 years, n (%)	29 (46.8)	16 (39.0)	10 (50.0)	0.647
Having an occupation, n (%)	33 (53.2)	23 (56.1)	13 (65.0)	0.653
Living together, n (%)	58 (93.5)	38 (92.7)	19 (95.0)	0.942
Presence of assistance from family members at the insulin injection, n (%)	32 (51.6)	11 (26.8)	3 (15.0)	0.003
Current smoker, n (%)	9 (14.5)	6 (14.6)	4 (20.0)	0.827
Current drinker, n (%)	14 (22.6)	17 (41.5)	8 (40.0)	0.090
Regular meal, n (%)	45 (72.6)	34 (82.9)	17 (85.0)	0.330
Exercise habit, n (%)	34 (54.8)	18 (43.9)	7 (35.0)	0.248
HbA1c (%)	7.9 (7.7–8.2)	7.7 (7.4–8.1)	7.6 (7.1–8.0)	0.316
eGFR (ml/min/1.73 m^2)	69.0 (63.8–74.2)	73.0 (66.6–79.4)	62.3 (53.1–71.4)	0.166
Total daily insulin (U/kg)	0.5 (0.4–0.5)	0.5 (0.4–0.6)	0.5 (0.4–0.6)	0.992
Intensive insulin therapy, n (%)	26 (41.9)	24 (58.5)	15 (75.0)	0.024

Continuous data were analyzed by analysis of covariance with adjustments for age and sex, and are shown as age- and sex-adjusted mean (95% confidence interval).

Dichotomous data were analyzed by χ^2 test and are shown as number (%).

HbA1c, hemoglobin A1c; eGFR, estimated glomerular filtration rate.
