# Supplementary material for: Predictors for Mild and Severe Hypoglycemia in Insulin-Treated Japanese Diabetic Patients
Source: PLoS One. 2015 Jun 23;10(6):e0130584. doi: 10.1371/journal.pone.0130584 (PMC4477874; doi:10.1371/journal.pone.0130584)
Supplement: S2 Table — Note: Continuous data were analyzed by analysis of covariance with adjustments for age and sex, and are shown as age- and sex-adjusted mean (95% confidence interval). Dichotomous data were analyzed by χ2 test, and are shown as number (%). HbA1c, hemoglobin A1c; eGFR, estimated glomerular filtration rate. (PDF) [file pone.0130584.s002.pdf]

S2 Table. Comparison between the characteristics of patients who were included and excluded from our analysis among all the patients who agreed to participate in the survey.

	**Patients who were included in the analysis**	**Patients who were excluded from our analysis**	***p* value**
n	123	62	
Age (years)	65.9 (63.6–68.3)	64.0 (60.7–67.3)	0.347
Men, n (%)	70 (56.9)	37 (59.7)	0.719
Type 1 diabetes, n (%)	25 (20.3)	14 (22.6)	0.641
Body mass index (kg/m^2)	25.0 (24.3–25.7)	24.0 (23.0–25.0)	0.115
Systolic blood pressure (mmHg)	136.3 (132.9–139.6)	134.2 (129.5–138.9)	0.468
Diastolic blood pressure (mmHg)	72.6 (70.9–74.4)	71.5 (69.1–74.0)	0.487
HbA1c (%)	7.8 (7.6–8.0)	7.6 (7.3–7.8)	0.131
eGFR (ml/min/1.73 m^2)	69.2 (65.5–73.0)	71.4 (66.0–76.7)	0.525

Continuous data were analyzed by analysis of covariance with adjustments for age and sex, and are shown as age- and sex-adjusted mean (95% confidence interval).

Dichotomous data were analyzed by χ^2 test, and are shown as number (%).

HbA1c, hemoglobin A1c; eGFR, estimated glomerular filtration rate.
